# Development and Characterization of an In Vitro Intestinal Model Including Extracellular Matrix and Macrovascular Endothelium

**DOI:** 10.1021/acs.molpharmaceut.3c00532

**Published:** 2023-09-07

**Authors:** Scarlett Zeiringer, Laura Wiltschko, Christina Glader, Martin Reiser, Markus Absenger-Novak, Eleonore Fröhlich, Eva Roblegg

**Affiliations:** † University of Graz, Institute of Pharmaceutical Sciences, Pharmaceutical Technology and Biopharmacy, Universitätsplatz 1, 8010 Graz, Austria; # Joanneum Research-Health, Neue Stiftingtalstraße 2, 8010 Graz, Austria; ‡ Research Center Pharmaceutical Engineering GmbH, Inffeldgasse 13, 8010 Graz, Austria; § Center for Medical Research, Medical University of Graz, Stiftingtalstraße 24, 8010 Graz, Austria; ⊥ BioTechMed-Graz, Mozartgasse 12/II, 8010 Graz, Austria

**Keywords:** in vitro intestinal model, hydrogel, extracellular matrix, endothelial cells, synthetic hydrogel

## Abstract

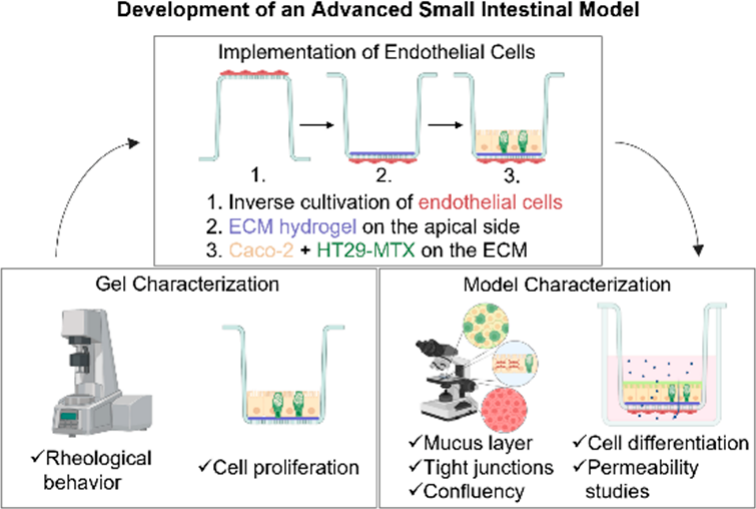

In vitro intestinal models are used to study biological processes, drug and food absorption, or cytotoxicity, minimizing the use of animals in the laboratory. They usually consist of enterocytes and mucus-producing cells cultured for 3 weeks, e.g., on Transwells, to obtain a fully differentiated cell layer simulating the human epithelium. Other important components are the extracellular matrix (ECM) and strong vascularization. The former serves as structural support for cells and promotes cellular processes such as differentiation, migration, and growth. The latter includes endothelial cells, which coordinate vascularization and immune cell migration and facilitate the transport of ingested substances or drugs to the liver. In most cases, animal-derived hydrogels such as Matrigel or collagen are used as ECM in in vitro intestinal models, and endothelial cells are only partially considered, if at all. However, it is well-known that animal-derived products can lead to altered cell behavior and incorrect results. To circumvent these limitations, synthetic and modifiable hydrogels (Peptigel and Vitrogel) were studied here to mimic xenofree ECM, and the data were compared with Matrigel. Careful rheological characterization was performed, and the effect on cell proliferation was investigated. The results showed that Vitrogel exhibited shear-thinning behavior with an internal structure recovery of 78.9 ± 11.2%, providing the best properties among the gels investigated. Therefore, a coculture of Caco-2 and HT29-MTX cells (ratio 7:3) was grown on Vitrogel, while simultaneously endothelial cells were cultured on the basolateral side by inverse cultivation. The model was characterized in terms of cell proliferation, differentiation, and drug permeability. It was found that the cells cultured on Vitrogel induced a 1.7-fold increase in cell proliferation and facilitated the formation of microvilli and tight junctions after 2 weeks of cultivation. At the same time, the coculture showed full differentiation indicated by high alkaline phosphatase release of Caco-2 cells (95.0 ± 15.9%) and a mucus layer produced by HT29-MTX cells. Drug tests led to ex vivo comparable permeability coefficients (*P*_app_) (i.e., *P*_app_; antipyrine = (33.64 ± 5.13) × 10^–6^ cm/s, *P*_app_; atenolol = (0.59 ± 0.16) × 10^–6^ cm/s). These results indicate that the newly developed intestinal model can be used for rapid and efficient assessment of drug permeability, excluding unexpected results due to animal-derived materials.

## 1 Introduction

The human small intestine can be divided into three sections, namely, the duodenum, jejunum, and ileum, and plays a central role in food digestion, nutrient absorption, and maintenance of homeostasis through interactions between host and microbes. From an anatomical perspective, the intestinal epithelium comprises a cell monolayer consisting of 90% absorptive enterocytes and 5% mucus-producing goblet cells. The remaining 5% are divided among enteroendocrine cells, tuft cells, and Paneth cells.^1^ The epithelium is bordered by an underlying extracellular matrix (ECM) in which the vasculature is embedded. As a noncellular component, the matrix comprises collagens, laminins, proteoglycans, and linker molecules and is in constant contact with cells through integrins (cell-surface receptors).^2^ All these contribute to the diverse functions of the ECM, such as structural support for cells, as well as cellular processes including differentiation, migration, and growth.^3^ There are two types of ECM, i.e., the interstitial matrix and the basement membrane. The interstitial matrix is located underneath the basement membrane, which in turn is a thin layer, about 100–400 nm thick, that underlies the epithelial cells and consists of four main components (i.e., Type IV collagen, laminin, nidogen, and perlecan).^4−6^ The functions include tissue separation, macromolecular filtering, structure support, and modulation of signaling processes through interactions with integrins.^2,7^ Another important feature of the small intestine is its high vascularization. Here, endothelial cells are the main component of the microvasculature lining blood and lymphatic vessels.^8^ They enable the delivery of absorbed substances such as nutrients, drugs, etc., to the liver and other organs and control the passage of antigens and commensal gut microbiota from the intestine into the bloodstream. Interestingly, recent studies have shown that 70 kDa particles cannot pass the intestinal-vascular barrier, suggesting that larger bacteria, for example, are filtered and cannot enter the bloodstream.^9^ This implies that the endothelium is also an important barrier system for particle systems, in addition to the functions described. Notably, *Shigella* spp. or *Listeria monocytogenes*, for example, have evolved specific mechanisms that lead to inflammatory responses and thus to disruption of the intestinal epithelium. This results in higher permeability and allows the bacteria to invade deeper intestinal tissues, potentially causing systemic infections.^10,11^ Apart from the barrier function, the endothelial cells are also involved in immunological processes by regulating the activation of immune cells through cytokine release and thus also indirectly influencing the epithelial cells.^12^

In recent years, in vitro models of the small intestine have become increasingly important for testing nutrient and drug absorption. To this end, cell-based in vitro models that accurately simulate human anatomy and physiology and simplify or facilitate the study of complex in vivo phenomena are being developed. They also create well-controlled and reproducible conditions for evaluating cell responses.^13^ Most two- (2D) and three-dimensional (3D) in vitro intestinal models comprise two main cell types, i.e., enterocytes (Caco-2 cells) and mucus-producing goblet cells (HT29-MTX cells), which are usually cultured for 21 days to achieve a confluent and fully differentiated cell model.^14^ Some more complex models also consider M cells, which account for approximately 10% of the epithelial cells and are a key player in phagocytosis and transcytosis of luminal macromolecules and antigens.^15^ A model taking these cell lines into account was developed by, e.g., Schimpel et al.^16^ Interestingly, the ECM is only partially or not at all considered in the development of in vitro models.^17^ Nevertheless, there are a few different approaches being tested to mimic the small intestine and consider the ECM. Most of these use scaffolds or hydrogels like collagen I or Matrigel.^14,18^ With respect to the latter, the components are extracted from Engelbreth-Holm-Swarm tumor cells from mice, as these tumor cells have a similar composition to that of the basement membrane. Matrigel contains collagen type IV, laminin, and nidogen, although many of the ingredients are not precisely identified. This may lead to unexpected results in experiments due to variations in the chemical and mechanical properties.^19,20^ The same applies to commonly used collagen I, which is mainly extracted from rat tails. Despite its origin, the collagen I hydrogel is often applied as a structural support in cell models and provides a 3D villi/crypt architecture or it is used as a scaffold which holds stromal cells such as fibroblasts, thus providing the tissue-structuring function of the ECM.^21,22^ However, with the use of animal-derived substances, it must be taken into account that xenogeneic substances can lead to altered cell behavior. Not only do the unpredictable rheological properties present a potential drawback, but the unknown composition also poses a particular risk, as, e.g., lactate dehydrogenase-increasing viruses can lead to immunological complications in cell culture. To overcome these problems, synthetic alternative hydrogels have recently gained importance.^23^ Synthetic hydrogels have the advantage that their composition and structure can be easily modified and thus their chemical and mechanical properties can be controlled. Additionally, bioactive substances such as adhesive peptides, ECM molecules (e.g., laminin, fibronectin, collagen), proteinases, or growth factors can be suspended in the gel to also allow the simulation and investigation of biochemical processes.^17^ Dosh et al., for example, synthesized two hydrogels (polyacrylamide hydrogels, i.e., L-pNIPAM, and L-pNIPAM-*co*-DMAc) and compared these to a common alginate hydrogel in cell culture experiments. It was shown that the intestinal cells formed the characteristic villus shape in 21 days, while differentiation was maintained upon cultivation on the synthesized hydrogel L-pNIPAM.^24^ Another approach was tested by Sung et al.^25^ Here, a villi/crypt-shaped mold was prepared and coated with collagen I or poly(ethylene glycol)-diacrylate (PEG-DA) hydrogels. Subsequent cultivation of Caco-2 cells for 21 days resulted in good proliferation and migration of cells and a confluent cell layer on the villi/crypt hydrogel scaffold.^25^ This was also the approach taken by Creff et al., who added fibronectin, an ECM glycoprotein, to the PEG-DA hydrogel. This led to improved Caco-2 cell adhesion, differentiation, and proliferation and once again underlines the importance of the ECM.^26^

Along with the ECM, the endothelium also plays an important role, as it functions as a barrier and regulates the tissue homeostasis through the interplay between endothelial, epithelial, and immune cells. This has been recently shown by Macedo et al.^27^ In this work, human pulmonary microvascular endothelial cells were cultured on the basolateral side of the insert, whereas intestinal cells were cocultured on a rat collagen I layer on the apical side, in which fibroblasts were embedded. This model was characterized in terms of differentiation and drug permeability while comparing two time points (i.e., 14 and 21 days). The results showed that endothelial cells influenced the contractility of fibroblasts by releasing specific proteins (e.g., ET-1) and thus possibly influencing the mechanical properties of the ECM. Furthermore, the additional endothelial cell layer led to decreased permeability values, compared to in vivo data and the intestinal coculture model without the endothelium. Finally, as for the assessment of the differentiation, the model was found to be fully differentiated only after 21 days.^27^

Although promising approaches have been presented, to our knowledge, there is no small intestinal static in vitro model that includes the ECM as well as the endothelium and is exclusively based on animal-free systems to exclude possible interactions.^28,29^ Hence, in this study starting from an already established Transwell coculture system consisting of Caco-2 and HT29-MTX cells, the first aim was to evaluate alternative xenofree materials to mimic the ECM. To this end, Matrigel, Peptigel, and Vitrogel were carefully studied with regard to their rheological properties, with the latter two being of animal-free origin. Additionally, cell proliferation studies were performed with the mono- and coculture to identify the most appropriate synthetic ECM candidate. Following these studies, endothelial cells were included in the model through basolateral cultivation by inverting a Transwell insert before the coculture was established on the ECM on the apical side. A careful characterization of the model was performed by testing the cell proliferation, determining the differentiation of Caco-2 cells (alkaline phosphatase (ALP) activity), and visualizing the mucus produced by HT29-MTX cells. Furthermore, transepithelial electrical resistance (TEER) measurements and occludin staining were performed to prove the integrity of the cell layer and the intact tight junctions. Finally, permeability studies were conducted using antipyrine, FITC-dextran, atenolol, and rhodamine123 (rho123). As the ECM reportedly has a positive effect on cell proliferation and differentiation, three time points (i.e., 7, 14, and 21 days) were examined and compared as part of the characterization to detect potentially accelerated differentiation of the model.

## 2 Materials and Methods

### 2.1 Materials

Dulbecco’s Modified Eagle’s Medium (DMEM), phosphate buffered saline (PBS; pH 7.4), 0.25% trypsin-ethylenediaminetetraacetic acid (trypsin-EDTA), fetal bovine serum (FBS), penicillin–streptomycin (Penstrep), and Hank’s Balanced Salt Solution (HBSS) were obtained from Gibco, Life Technologies Corporation (Painsley, United Kingdom) and used for cell culture experiments. MEM Nonessential Amino Acid Solution (100×; NEAA), Bovine Serum Albumin (BSA), and Acridine Orange (AO) were purchased from Sigma-Aldrich (Munich, Germany). HyClone (DMEM without phenol red) was obtained from GE Healthcare Life Sciences (Logan, UT, USA). Alexa Fluor 488 Phalloidin, Hoechst 33342, and primary occludin monoclonal antibody were obtained from Thermo Fisher Scientific (Vienna, Austria). Alexa Fluor 568 goat anti-mouse IgG1 was purchased from Thermo Fisher Scientific (Waltham, MA, USA).

### 2.2 Cell Culture

Caco-2 cells (ACC169, clone of the German collection of microorganisms and cell cultures), kindly provided by E. Fröhlich (Medical University of Graz, Austria), were cultivated in DMEM, supplemented with 10% FBS, 1% Penstrep, and 1% NEAA, at 37 °C and 5% CO_2_ water-saturated atmosphere. Mucus-producing HT29-MTX cells, provided by Dr. Thécla Lesuffleur (Paris, France), and the endothelial cell line EA.hy926 (provided by A. Kungl, University of Graz, Austria) were cultured under the same conditions. The medium was changed every second to third day, and subcultivation was performed once a week after reaching confluency, using trypsin-EDTA 0.25%.

### 2.3 Characterization of Synthetic Alternatives to Matrigel

To find alternatives to the animal-derived Matrigel (Corning, Phoenix, AZ, USA), synthetic hydrogels, namely, Peptigel (Manchester BIOGEL, Manchester, United Kingdom) and Vitrogel (Peprotech, Hamburg, Germany), were characterized with regard to their rheological behavior and their impact on cell proliferation.

The rheological measurements included the determination of dynamic viscosity, shear and loss modulus (*G*′ and *G*″), and thixotropy. First, the linear viscoelastic region (LVE) was measured to define the range of the applied strain in which the internal structure of the gels is not destroyed. Therefore, a deformation range (γ) between 0.01% and 100% (log) at an angular velocity (ω) of 10 rad/s was tested. Based on these results, a deformation (γ) of 1% was selected for the frequency sweep measurements at an angular velocity (ω) between 0.1 and 100 rad/s to determine *G*′ and *G*″. In addition to this, the dynamic viscosity η was measured at a shear rate (γ̇) range between 0.1 and 100 s^–1^ (log). For the measurements of the LVE, *G*′ and *G*″ and the dynamic viscosity η, 25 measurement points were used. The determination of the thixotropy was composed of three sections. The first section comprised 10 measurement points and was performed at a shear rate (γ̇) of 0.5 s^–1^. The shear rate (γ̇) of the second section was increased to 250 s^–1^ for 10 measurement points for 90 s and for the third section decreased again to 0.5 s^–1^ for 50 measurement points. For these measurements, 80 μL of each gel were pipetted into a prefabricated circular mold (diameter 8 mm). Incubation was performed at 37 °C for 30 min to induce gelation, using a plastic cap to prevent evaporation. Matrigel and Peptigel were used undiluted, Vitrogel was mixed with 20% serum-free cell culture medium according to the manufacturer’s instructions. For all rheological measurements, a rotational viscometer with a PP08 system was used (Physica MCR 301 Rotational viscometer, Anton Paar, Graz, Austria).

#### 2.3.1 Cell Proliferation

The cell proliferation on Matrigel, Vitrogel, and Peptigel was determined using the CellTiter 96 Aqueous Nonradioactive Cell Proliferation Assay (Promega Corporation, Madison, WI, USA) according to the manufacturer’s protocol. Briefly, a 96-well plate (Cellstar, Greiner Bio-One GmbH, Friedrichshafen, Germany) was coated with Matrigel (20 μL), Peptigel (40 μL), and Vitrogel (40 μL, diluted 1:1 with Vitrogel Dilution Solution) according to the manufacturer’s instructions. In the course of coating, it was additionally checked whether 40 μL of Vitrogel completely covered the well surface. For this purpose, the gel was stained with methylene blue solution (Lactan, Graz, Austria) and photographed afterward. After this, the desired volume of the gels was added to the well, followed by an incubation time of 30 min at 37 °C to enable the gel to solidify. Afterward, Caco-2 cells (1 × 10^4^ cells/well) were seeded on coated and blank wells as a cell control. Cells were cultured for 7 days before the assay was performed. Briefly, cells were washed once with HBSS, followed by a 4 h incubation with the MTS reagent. Finally, the absorbance measurement was performed at 490 nm using a microplate reader (CLARIOstar^Plus^, BMG LABTECH, Ortenberg, Germany).

### 2.4 Establishing the Cell Culture Model

#### 2.4.1 Inverse Cultivation of Endothelial Cells on the Basolateral Side

For the inverse cultivation of the endothelial cell line EA.hy926, inserts of a 24-well Transwell plate (Thincert, Greiner Bio-One GmbH, Friedrichshafen, Germany) were placed upside down in a 12-well plate (Cellstar, Greiner Bio-One GmbH, Friedrichshafen, Germany) to enable the addition of the cells (1 × 10^4^ cells/well) on the basolateral side of the filter. After 2 h incubation at 37 °C and 5% CO_2_ saturation, the inserts were placed back in the original plate and used for further studies.

To confirm a successful cultivation of the endothelial cells, cell visualization and TEER measurements were performed. For the cell visualization, cells were stained as reported by Schimpel et al.^16^ Briefly, cells were fixed and permeabilized, followed by a 25 min incubation period with 1 U of Alexa Fluor 488 Phalloidin and 24 μg/mL of Hoechst 33342 to stain the cell skeleton and the cell nuclei, respectively. Filters were then cut, placed, and mounted on glass slides to enable the microscopy with a confocal light scanning microscope LSM 510 Meta (cLSM; Carl Zeiss GmbH, Vienna, Austria) equipped with a ZEN2008 software package using Axio Observer (Zeiss; camera: Axio Cam) at λ_Ex_ = 488 nm for the green channel and λ_Ex_ = 405 nm excitation for the blue spectral region. The TEER measurements were performed after 7 days using an EVOM Manual High Throughput Screening System (World Precision Instruments, Sarasota, FL, USA).

#### 2.4.2 Apical Cultivation of a Caco-2/HT29-MTX Coculture on Vitrogel

**Figure 1:**
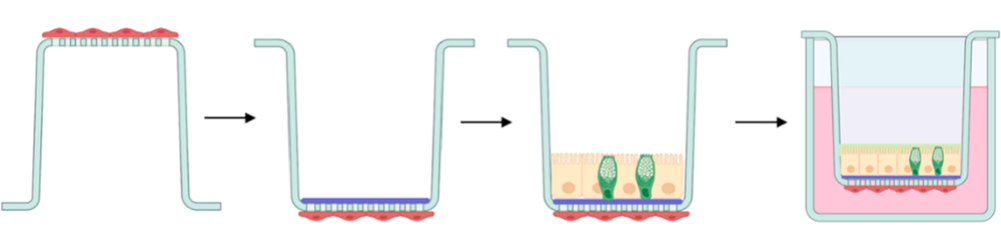
Establishment of the in vitro intestinal model starting with the inverse cultivation of endothelial cells, adding of the hydrogel on the apical side followed by cultivation of the coculture. Created with BioRender.com.

After the inverse cultivation of endothelial cells, the apical side of the insert was coated with 40 μL of Vitrogel, followed by an incubation time for 30 min at 37 °C to enable the gel to solidify. A coculture consisting of Caco-2 and HT29-MTX cells (1.5 × 10^5^ cells/well) in a 7:3 ratio was then added onto the gel (Figure 1). This configuration, i.e., Caco-2/HT29-MTX coculture-Vitrogel-endothelium, is referred to as “model”. In parallel, Caco-2 cells, HT29-MTX cells, and a coculture thereof were cultured as cell controls in blank inserts without gel. The model and cell controls were cultured for 7, 14, and 21 days, and medium was changed three times a week.

As a next step, TEER values and cell proliferation were determined after 7, 14, and 21 days in the model. A Caco-2 monoculture and a Caco-2/HT29-MTX coculture seeded on uncoated inserts were used as cell controls. The MTS assay was performed in the same manner as described earlier, except that 100 μL of the substrate/HBSS mixture was transferred to a 96-well plate (Cellstar, Greiner Bio-One GmbH, Friedrichshafen, Germany) after the incubation time of 4 h to enable the absorbance measurement with the CLARIOstar^Plus^.

### 2.5 Histochemical Characterization

#### 2.5.1 Immunofluorescence Staining

On day 7, 14, and 21 the model and cell controls (Caco-2 monoculture and Caco-2/HT29-MTX coculture) were fixed, permeabilized, and blocked according to the manufacturer’s instructions. For the visualization of the tight junctions, the cells were incubated with the primary antibody against occludin at a concentration of 2.5 μg/mL in 0.1% BSA for 3 h at room temperature (RT), followed by additional three washing steps. After a 45 min incubation at RT with the secondary antibody (4 μg/mL in 0.2% BSA; Alexa Fluor 568 goat anti mouse IgG1) and three times washing, Alexa Fluor 488 Phalloidin (1 U) and Hoechst 33342 (24 μg/mL) were added, incubated for 25 min at RT, and again washed three times with PBS. As a last step, the filters of the inserts were removed, placed, and mounted on a slide. Microscopy was performed either with a cLSM or a Nikon Eclipse Ti2Microscope equipped with Andor Zyla sCMOS camera at λ_Ex_ = 488 nm for the green channel, λ_Ex_ = 405 nm excitation for the blue spectral region, and λ_Ex_ = 543 nm for the orange channel.

#### 2.5.2 Cell Differentiation of Caco-2 Cells

The differentiation of Caco-2 cells was determined by measuring the ALP release. Therefore, SIGMAFAST *p*-nitrophenyl phosphate (pNPP) tablets (Sigma-Aldrich, Vienna, Austria) were used. The release was tested in the cell model and cell control (Caco-2 monoculture and Caco-2/HT29-MTX coculture) after 7, 14, and 21 days of cultivation. Briefly, 150 μL of the reaction buffer (*c*_pNPP_ = 1 mg/mL, *c*_Tris_ = 0.2 M in H_2_O dest.) were added into the apical compartment of each Transwell insert and incubated for 30 min at RT under light exclusion. 100 μL of each well were then transferred into a 96-well plate and absorbance was measured at 409 nm with a UV–vis plate reader (CLARIOstar^Plus^, BMG LABTECH, Ortenberg, Germany).

#### 2.5.3 Visualization of Mucus Layer Produced by HT29-MTX Cells

To confirm a confluent mucus layer, AO was used to visualize the mucus produced by HT29-MTX cells on days 7, 14, and 21. Therefore, the model and cell control (HT29-MTX monoculture and Caco-2/HT29-MTX coculture) were once washed with HBSS followed by adding 20 μL/well of an AO solution (20 μg/mL in HBSS) and an incubation for 10 min at 37 °C. The inserts were washed two times with HBSS, and the filters were removed and mounted on glass slides. Fluorescence microscopy (λ_Ex_ = 525 nm (green) and 650 nm (red)) was performed with the cLSM.

### 2.6 Drug Transport Studies

For permeability assays, FITC-dextran (avg mol wt 150 000 Da; Polysciences Europe GmbH, Eppelheim, Germany), antipyrine (Sigma-Aldrich, Vienna, Austria), rho123 (Biotium, Fremont, CA, USA), and atenolol (GL Pharma, Lannach, Austria) were used as model drugs. The permeability was tested from the apical to basolateral (A → B) and in the case of rho123 and atenolol also from the basolateral to apical (B → A) side. The measurements were performed in the model, Caco-2 monoculture and Caco-2/HT29-MTX coculture after 14 and 21 days. As transport buffer, Krebs-Ringer bicarbonate buffer (Sigma-Aldrich, Vienna, Austria) was used in all cases. To test the A → B permeability, 300 μL samples of the drug solutions were added to the apical side and 600 μL drug solution was added to the basolateral side to test B → A permeability. The same drug concentration was used for both directions. For the assays, the cells were washed with HBSS prior to adding the drug solutions. Further, TEER was measured (World Precision Instruments, Sarasota, FL, USA) before and after the assay. FITC-dextran was applied at a concentration of 0.4 mg/mL;^30^ antipyrine and atenolol were tested at a concentration of 1.88 and 0.2 mg/mL, respectively.^16^ Rho123 was used at a concentration of 1.9 μg/mL. At predetermined time points (15, 30, 45, 60, 90, 120, 150, and 180 min) the buffer of the acceptor compartment was completely removed and replaced with fresh, prewarmed Krebs-Ringer bicarbonate buffer. FITC-dextran and rho123 were quantified using the CLARIOstar^Plus^ by fluorescence measurements at λ_Ex_ = 492 nm/λ_Em_ = 518 nm and λ_Ex_ = 485 nm/λ_Em_ = 520 nm, respectively. Quantification of antipyrine and atenolol was evaluated by high performance liquid chromatography (HPLC) using an Agilent 1260 Infinity II system. For this, the system was equipped with a model series 1260 DAD (G4212B) detector, a 1260 QuatPump (G7111B) elution pump, a 1260 vial sampler (G7129A), and a 1260 MCT (G7116A) column heater/cooler. For separation of antipyrine, a C18 MOS-Hypersil 120 Å column (250 × 4.0 mm, 5 μm; VDS optilab, Berlin, Germany) was used at a temperature of 35 °C. The analysis was performed at 230 nm. The mobile phase for chromatographic separation consisted of 58% (v/v) Milli-Q water adjusted to pH 3.0 with trifluoroacetic acid and 42% (v/v) of an acetonitrile:methanol mixture (50:50; v/v). The separation was performed in an isocratic mode with flow rate of 1 mL/min, and an injection volume of 2 μL. For the standard curve, an antipyrine stock solution (18.8 mg/mL) was prepared, which was subsequently diluted to the desired concentrations (0.0188–1.88 mg/mL) with Krebs-Ringer bicarbonate buffer. To determine the linearity of the method, the detector response was plotted against the drug concentration from the standard curve. The LLOQ for antipyrine equaled 2 × 10^–5^ mg/mL. Atenolol samples were separated using a ZORBAX 300 Extend C18 column (4.6 × 100 mm, 3.5 μm) at 25 °C; detection was performed at 220 nm. As mobile phase, a phosphate buffer (pH 6.2):methanol mixture in a ratio of 9:1 (v/v) was used. The measurement was performed in an isocratic mode with a flow rate of 0.7 mL/min and 20 μL injection volume. The calibration curve for atenolol was measured in a concentration range between 2 × 10^–4^ mg/mL and 0.2 mg/mL. The LLOQ was determined as described earlier and resulted in 2 × 10^–4^ mg/mL. Chromatography Data Station software (OpenLAB CDS, ver C.01.06) was applied for data acquisition. Based on the data obtained from the HPLC-UV measurements, the apparent permeability coefficient (*P*_app_, cm/s) for all drugs was calculated using the following equation:^31^
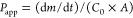
where d*m*/d*t* is the amount of drug (mg) permeated into the acceptor compartment over time (s), *A* is defined as the contact area of the insert (i.e., 0.33 cm^2^), and *C*_0_ is the initial drug concentration in the donor compartment (mg/mL).

### 2.7 Statistical Analysis

Results presented are expressed as mean values ± standard deviation (SD). For statistical analysis of the data, a Student’s *t* test was performed, and differences were considered significant at levels of *p* ≤ 0.05 (*), *p* ≤ 0.01 (**), and *p* ≤ 0.001 (***).

## 3 Results

### 3.1 Characterization of the Gels

#### 3.1.1 Rheological Characterization

**Figure 2:**
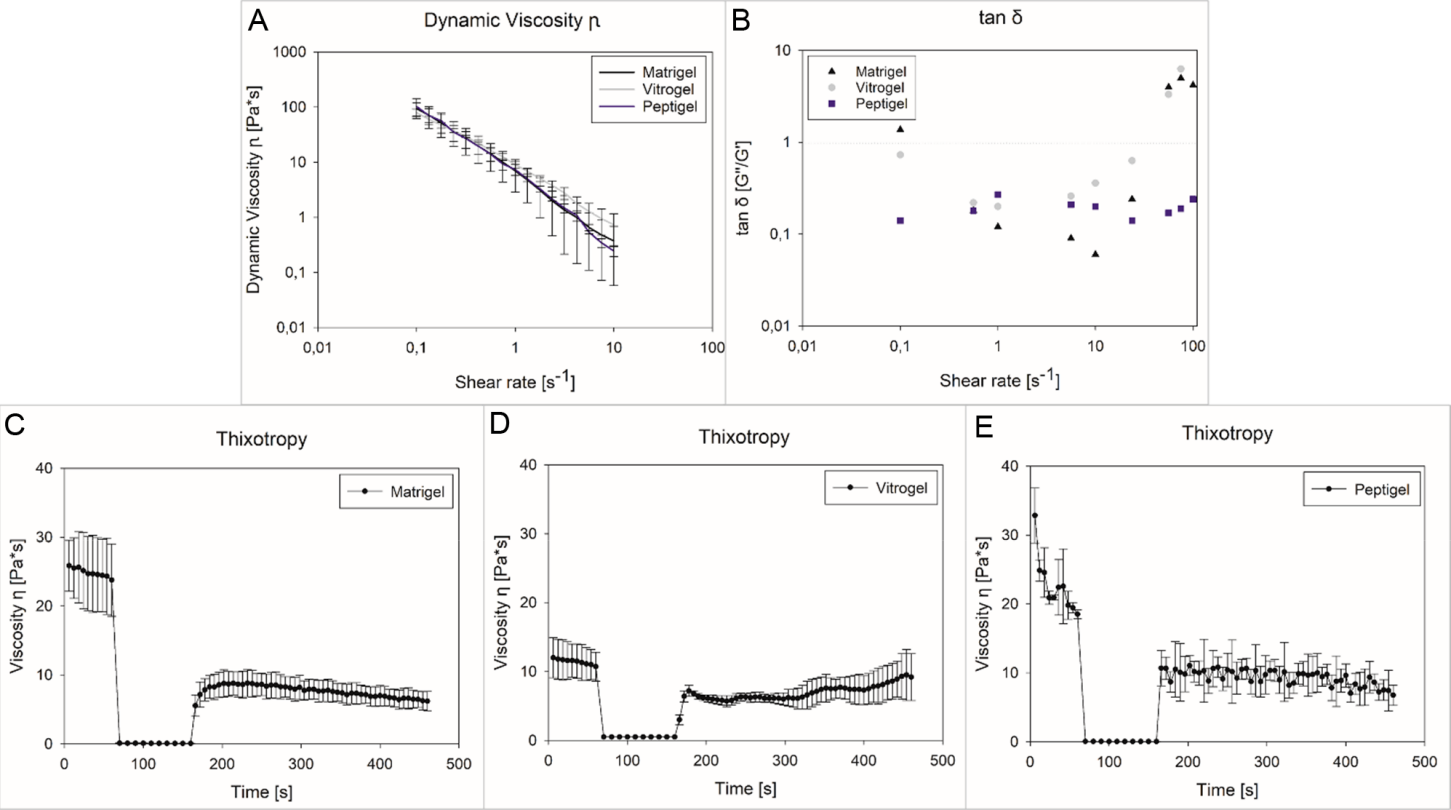
Rheological characteristics of Matrigel, Vitrogel, and Peptigel. (A) Dynamic viscosity η with changing shear rates (γ̇). (B) depicts tan δ, describing the gels behavior as viscous liquid or elastic solid. (C–E) shows the thixotropic curve of the three gels, indicating the rebuilding of the inner gel structure in all three cases.

The rheological measurements show a shear thinning behavior of all tested gels, i.e., Matrigel, Peptigel, and Vitrogel. The dynamic viscosity η of Matrigel, Vitrogel, and Peptigel at a shear rate (γ̇) of 0.1 s^–1^ is 93.9 ± 25.0, 79.6 ± 12.4, and 102.8 ± 40.5 Pa s, respectively (Figure 2A). Above a shear rate (γ̇) of 7.5 s^–1^, all viscosity values are below 1.0 Pa s, indicating a destroyed internal structure and shear-thinning behavior of the gels. The determination of *G*′ and *G*″ and the resulting loss factor tan δ, defined as *G*″/*G*′,^32^ was also conducted. Thereby, tan δ describes the viscoelastic behavior of the matrix; more precisely, if tan δ < 1, the gel acts as an elastic solid, and if tan δ > 1, it behaves like a viscous liquid. All gels showed a tan δ < 1 at shear rates (γ̇) between 0.1 s^–1^ and 29.0 s^–1^, which corresponds to an elastic solid. Above a shear rate (γ̇) of 30.0 s^–1^, tan δ for Vitrogel and Matrigel was >1, indicating destruction of the internal structure and liquefaction of the gels (Figure 2B). To further determine the thixotropic behavior of the gels, the recovery of the internal structure after applying shear forces was determined. After complete destruction of the internal network (viscosity η < 0.1 Pa s), all gels recovered. The internal structure recovery of Vitrogel reached 78.9 ± 11.2%. In comparison, 42.8 ± 0.3% of the Peptigel and 30.2 ± 11.7% of the Matrigel were reconstituted (Figure 2C–E).

#### 3.1.2 Cell Proliferation

**Figure 3:**
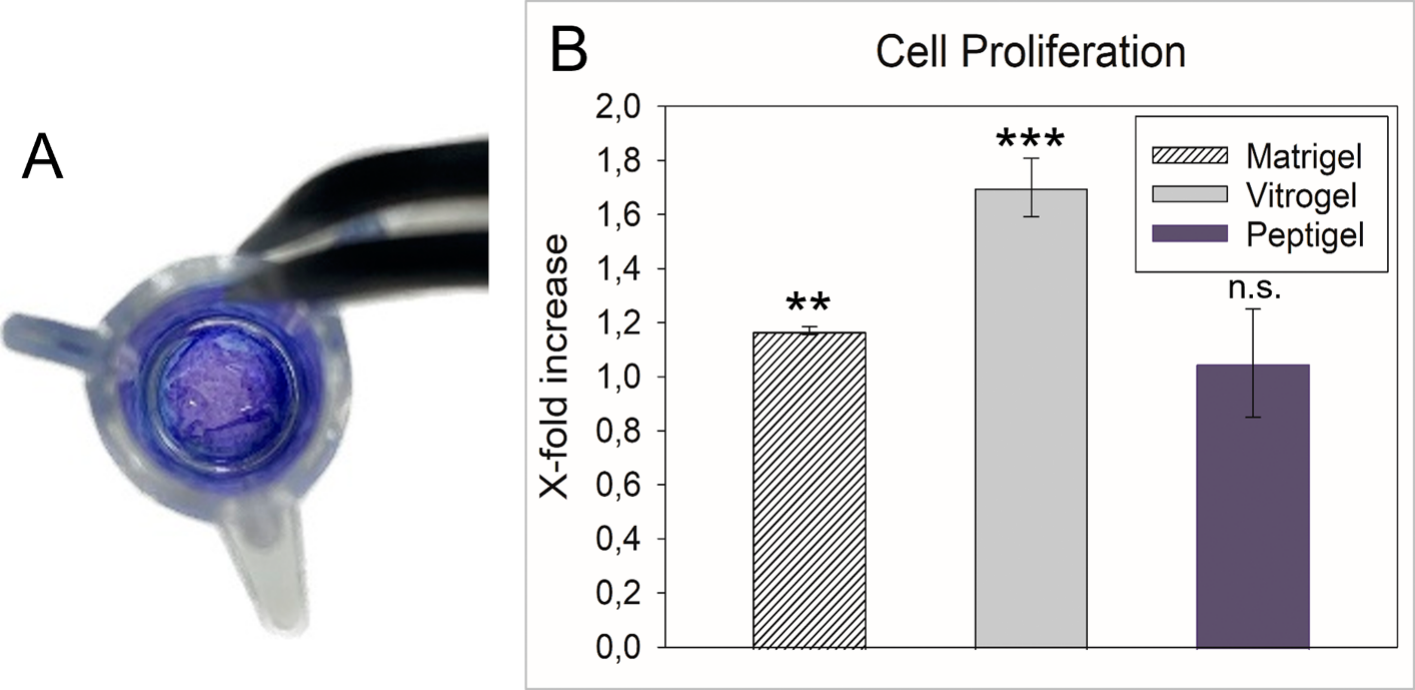
(A) 40 μL of Vitrogel, stained with methylene blue solution, covers the entire area of a common 24-well Transwell insert. (B) Cell proliferation analysis after 7 days of cultivation of Caco-2 cells on hydrogels. Vitrogel increased cell proliferation by a factor of 1.70 compared to a cell control.

In addition to the rheological characterization, cell proliferation of Caco-2 cells was studied on Matrigel-, Peptigel-, and Vitrogel-coated wells. Prior to this, methylene blue staining of the gel was performed. The results confirmed that 40 μL of the Vitrogel entirely covered the filter surface of the insert and were therefore used for all further experiments (Figure 3A). The obtained results were compared to those of a cell control in blank wells (Figure 3B). It was found that after 7 days, the cells grown on Peptigel exhibited a similar cell proliferation rate as the cell control (i.e., 1.05 ± 0.20), while cells cultured on Matrigel showed a slight increase by a factor of 1.17 ± 0.02. By contrast, cells cultured on Vitrogel further increased cell proliferation by a statistically significant factor of 1.70 ± 0.11 (***p ≤ 0.001). Thus, Vitrogel was identified as the most appropriate ECM candidate and used for further experiments.

### 3.2 Development of the In Vitro Cell Culture Model

#### 3.2.1 Inverse Cultivation and Apical Cultivation of Coculture

**Figure 4:**
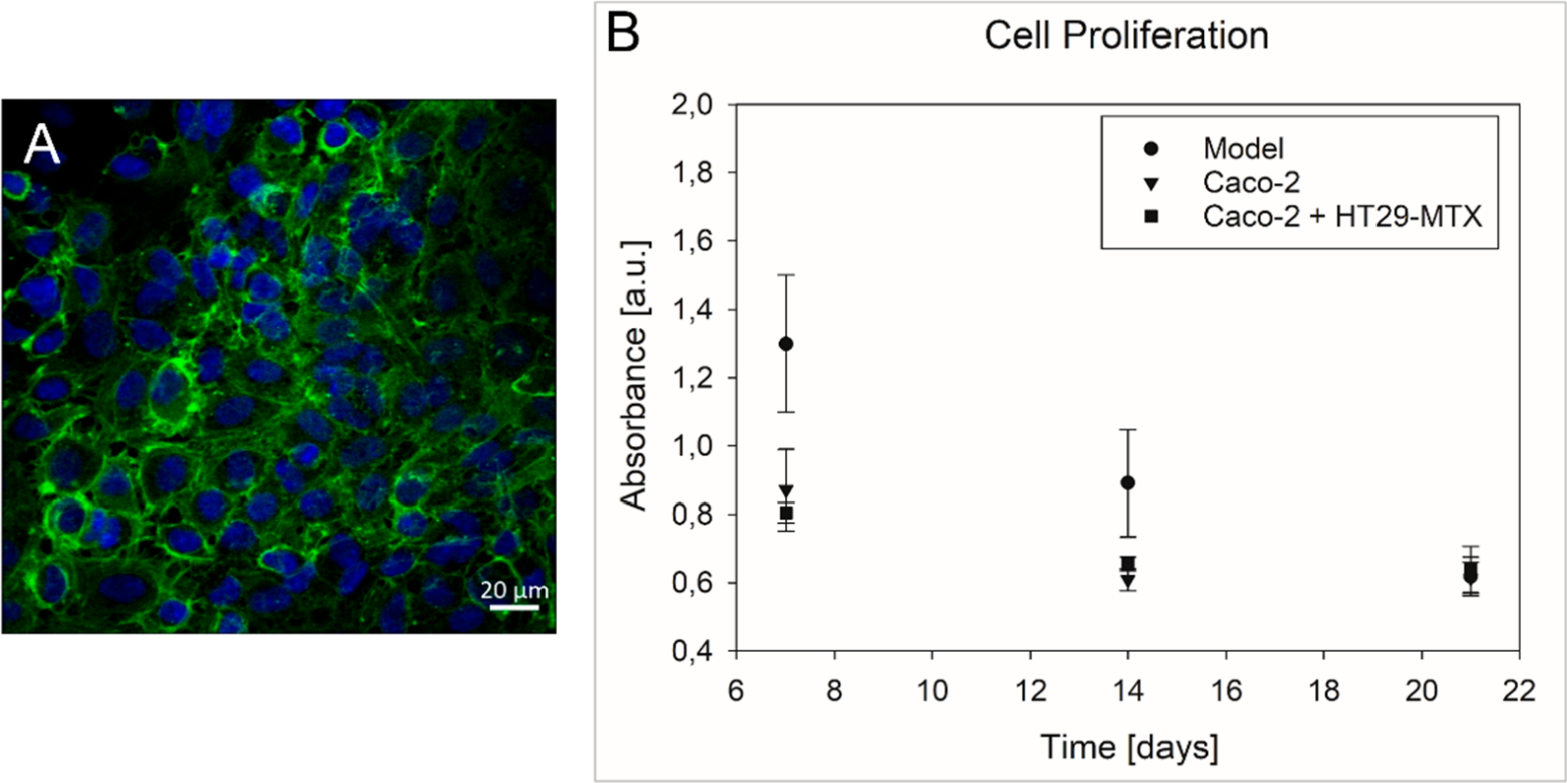
(A) A confluent endothelial cell layer was formed after 7 days of cultivation on the basolateral side of the insert. Nuclei are shown in blue (λ_Ex_ = 405 nm); the cytoskeleton is shown in green (λ_Ex_ = 488 nm). (B) The cell proliferation studies in the model and in blank wells showed that the coculture in the model exhibits higher cell proliferation on days 7 and 14, reaching similar results on day 21 as the cultivation in blank wells.

Endothelial cells EA.hy926 were cultured using inverse cell cultivation. After 2 h, cells adhered to the basolateral side, which was confirmed via TEER measurements and visualization. The cultivation for 7 days revealed TEER values of 15.7 ± 1.9 Ω cm^2^, which coincides with the literature (i.e., 14.0–23.7 Ω cm^2^).^33^ These results were confirmed by fluorescence microscopy. Figure 4A shows a confluent EA.hy926 cell layer on the basolateral side of a Transwell insert after 7 days cultivation.

The applicability of the model for long-term cultivation of the coculture was studied with respect to cell proliferation after 7, 14, and 21 days with and without Vitrogel. Figure 4B shows the absorbance measurement results representing cell proliferation. The data obtained for cells grown on Vitrogel showed significantly higher absorption values on day 7 (***p ≤ 0.001) and 14 (**p ≤ 0.01) (i.e., 1.299 ± 0.200 and 0.891 ± 0.157) compared to the mono- and coculture grown on uncoated wells (0.871 ± 0.119, 0.609 ± 0.032 and 0.805 ± 0.029, 0.655 ± 0.020, respectively). On day 21, similar values for all cell cultures ranging from 0.614 to 0.639 were obtained (Figure 4B).

TEER measurements of the coated and uncoated cell cultures showed similar results. On day 7, all cultures exhibited values below 115.6 Ω cm^2^ suggesting a non-confluent cell layer. After 14 days, the TEER values increased to 226.4 ± 19.9 Ω cm^2^ for the model, 358.6 ± 53.6 Ω cm^2^ for the Caco-2 monoculture, and 269.4 ± 9.7 Ω cm^2^ for the coculture. By day 21, TEER values of 332.3 ± 26.0 Ω cm^2^, 426.6 ± 15.2 Ω cm^2^, and 376.7 ± 41.3 Ω cm^2^ were reached.

#### 3.2.2 Characterization of the Model

When fully differentiated, Caco-2 cells exhibit brush borders which in turn secrete specific enzymes.^34^ To determine if the Caco-2 cells are fully differentiated in the model, the activity of the brush border enzyme ALP was measured on days 7, 14, and 21. Caco-2 monocultures were used as cell control. In general, a lower ALP release was obtained for all cultures grown on untreated wells compared with the treated ones. Regarding the latter, the HT29-MTX monoculture did not exceed an ALP release above 34.8 ± 4.4%. The model reached its maximum absorbance, i.e., 95.0 ± 15.9% on day 14, while on day 21, 83.0 ± 1.3% ALP release was determined. By contrast, the coculture grown on untreated wells reached an ALP release of 61.1 ± 8.6% after 14 days and only 89.6 ± 10.6% on day 21. The reduced level of ALP can be explained by the lower number of Caco-2 cells in the coculture and the formation of the mucus layer, as also presented by Schimpel et al.^16^

**Figure 5:**
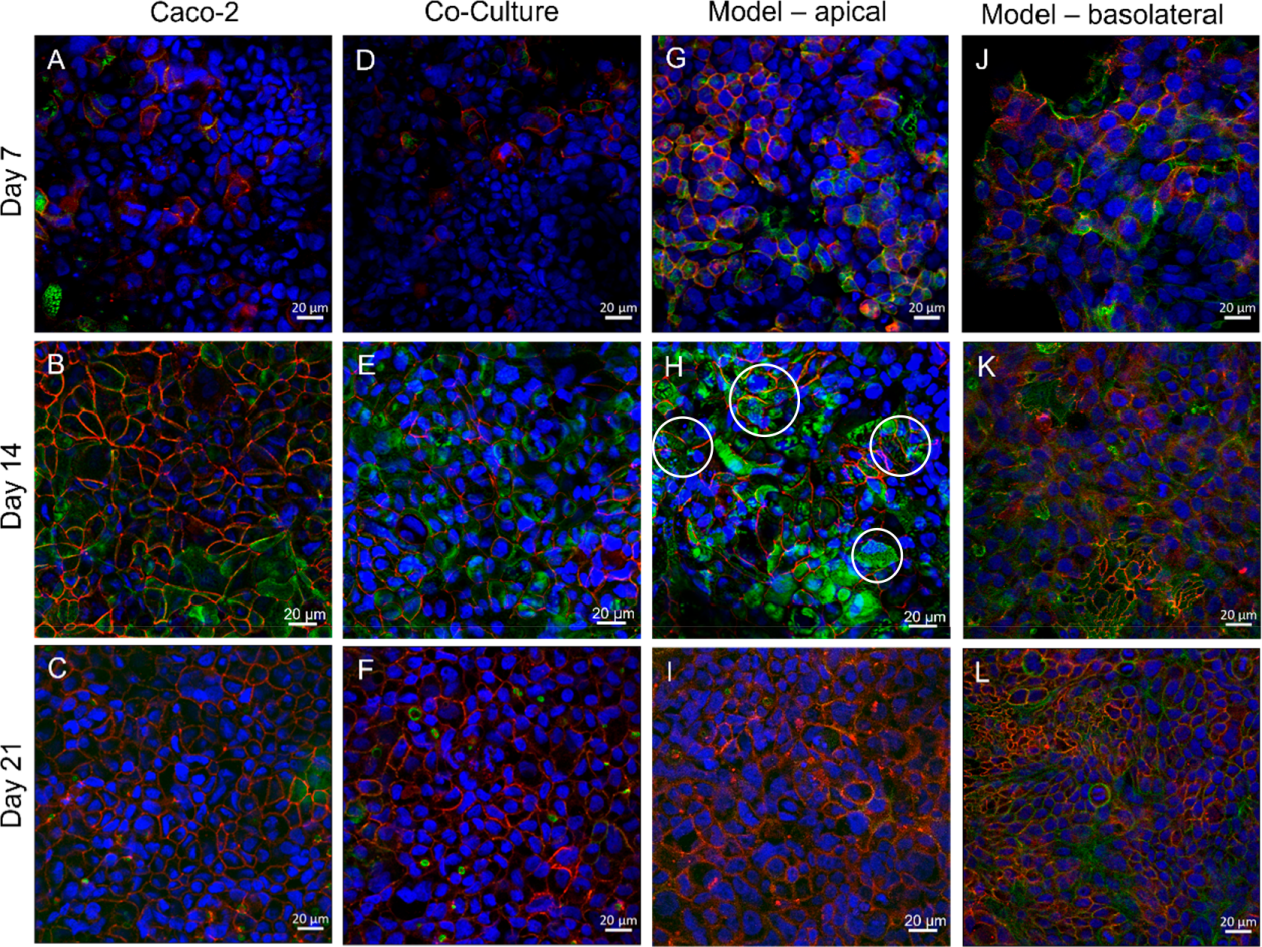
Visualization of tight junctions over the course of 21 days: a comparison of the tight junction formation in Caco-2 monoculture (A–C), Caco-2/HT29-MTX coculture (D–F), and the model (G–I) is illustrated; (J–L) shows tight junctions in the basolateral endothelial cell layer. Cell nuclei were stained in blue (λ_Ex_ = 405 nm), cytoskeleton in green (λ_Ex_ = 488 nm), and occludin in red (λ_Ex_ = 543 nm). The white circles indicate the microvilli structure of Caco-2 cells.

Another indicator of a differentiated and fully developed cell culture model is the formation of tight junctions. These cell–cell contacts provide a barrier function in the intestine and regulate paracellular permeability.^35^ To prove the presence of tight junctions, the transmembrane protein occludin was visualized through immunostaining. Figure 5 displays microscopic images of a Caco-2 monoculture (A–C) and a coculture (D–F) grown in blank wells and the model viewed both from the apical (G–I, Caco-2/HT29-MTX coculture) and basolateral sides (J–L, EA.hy926 cell culture). Images were taken on days 7, 14, and 21. On day 7, a nonconfluent cell layer with isolated tight junctions was found for all samples (A, D, G, J). At day 14, the model showed a confluent cell layer connected by tight junctions (H). In addition, a regular brush border with well distributed microvilli typical for Caco-2 cells was evident (indicated by the white circles in Figure 5H). Images taken from the basolateral side confirmed a confluent endothelium exhibiting tight junctions on days 14 and 21 (K,L).

**Figure 6:**
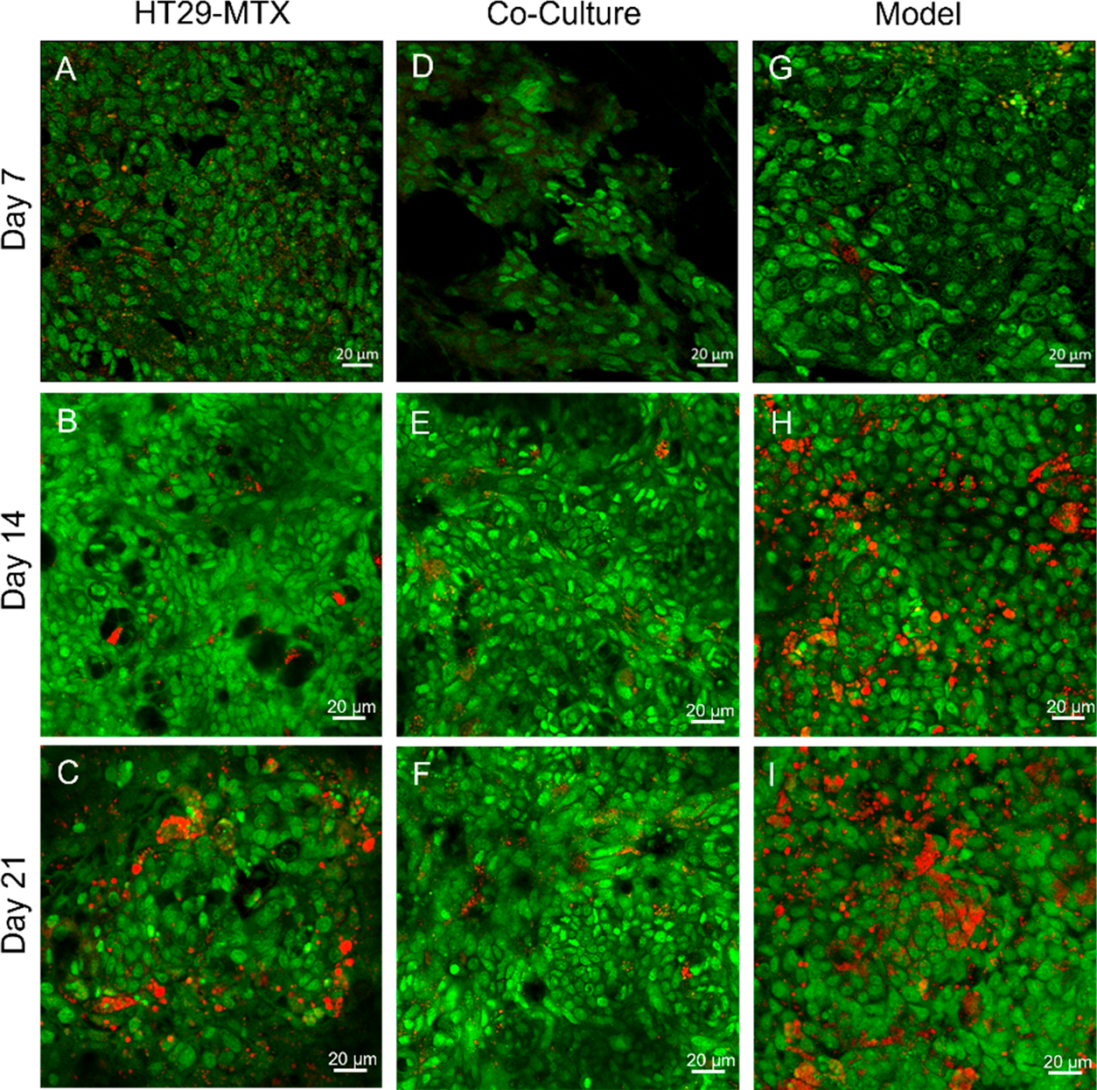
Mucus staining using AO. The figure illustrates mucus in red (λ_Ex_ = 650 nm), produced by the goblet cell line HT29-MTX, and the cells in green (λ_Ex_ = 525 nm).

The third indicator for a fully developed cell culture model is the formation of a mucus layer that covers the enterocytes. Therefore, AO was used as a staining agent for the coculture grown without gel and the model and compared to a HT29-MTX monoculture cell control (Figure 6). Since AO is a pH-sensitive substance, the emission shifts to the red spectrum upon contact with the acidic mucus leading to a red staining of the mucus and a green staining of the cells. As expected, the Caco-2 monoculture did not secrete significant amounts of mucus compared to the cell control. For HT29-MTX cells, mucus production increased over the progression of time (Figure 6A–C). Mucus production in the model was similar to the HT29-MTX monoculture on day 7, but it increased significantly on day 14 so that a complete layer was formed. These results are comparable to day 21. By contrast, the coculture in the blank wells did not show a confluent mucus layer within the time tested.

### 3.3 Drug Transport Studies

To further characterize the developed in vitro model, permeability studies were performed. To this end, different substances were used, such as FITC-dextran, a non-permeable substance, atenolol, a moderately permeable substance, as well as antipyrine, a highly permeable substance, and rho123, an efflux-mediated drug.^36^ The permeability studies were performed after 14 and 21 days of cultivation (Table 1). The data were compared with data found in the literature.^30,37−39^ For FITC-dextran no *P*_app_ coefficient could be determined. The values obtained were below the LLOQ, which indicates that the permeability is below 1%. In contrast, antipyrine resulted in *P*_app;A→B_ values of (33.64 ± 5.13) × 10^–6^ cm/s for day 14 and (29.34 ± 3.31) × 10^–6^ cm/s for day 21. In the case of atenolol, two *P*_app_ values were determined (Table 1) (i.e., *P*_app;A→B_ and *P*_app;B→A_), resulting in higher *P*_app;B→A_ values than those for the A → B direction. This suggests an efflux-mediated transport of the drug candidate. Finally, due to the low permeation of rho123 in the A → B direction, no *P*_app_ values could be evaluated; on the contrary, *P*_app;B→A_ values of (2.39 ± 0.43) × 10^–6^ cm/s (day 14) and (3.22 ± 0.47) × 10^–6^ cm/s (day 21) were obtained.

**Table 1:** Apparent Permeability Coefficients (*P*_app_; ×10^–6^ cm/s) of Antipyrine, Atenolol, and Rho123 Obtained in the Model After 14 and 21 Days and in A → B and B → A Directions

	antipyrine		atenolol		rho123	
	day 14	day 21	day 14	day 21	day 14	day 21
*P*_app;A→B_	33.64 ± 5.13	29.34 ± 3.31	0.59 ± 0.16	0.55 ± 0.61	<LOD	<LOD
*P*_app;B→A_	N.D.	N.D.	4.30 ± 0.07	1.80 ± 0.36	2.39 ± 0.43	3.22 ± 0.47

## 4 Discussion

The ECM and the proteins which come with it have a profound influence on the cell differentiation and proliferation in the human intestinal tissue.^40^ Due to this, many research groups include different types of scaffolds in in vitro cell culture to mimic the ECM. To this day, many of the newly developed in vitro models use hydrogels such as rat collagen type I or Matrigel as a structural basis underlying epithelial cell layers.^41^ Matrigel for example is a basement membrane extract from animal origin, as part of the ECM. However, due to their animal origin, the resemblance to human occurrences is often not provided. Aisenbrey et al. have reported that Matrigel is often affected by batch-to-batch variability, which not only affects the composition but also leads to different results in terms of mechanical and biochemical properties, resulting in a lack of reproducibility in experiments.^23^ Similar findings were reported also for animal-derived collagen products by Maji et al.^41^ However, not only do the components of the hydrogels have an influence on the cells; their rheological properties also lead to differences in, e.g., cell migration.^42,43^ These aspects point out the importance of synthetic, xenofree matrices, enabling the development of tissue specific compositions and rheological properties, simulating the ECM.

To the best of our knowledge, no detailed rheological data of in vivo intestinal ECM are described in the literature. Nevertheless, the rheological characterization of the gels is essential to compare them with known in vivo parameters. All gels showed shear-thinning properties above a shear rate (γ̇) of 7.5 s^–1^ (without significant differences), while the obtained values for tan δ are indicative of viscoelastic gels. This leads to the assumption that the samples would withstand the physiological pressure in the intestine, exerted by peristaltic motility, which accounts for a shear rate (γ̇) of 0.15 s^–1^.^44^ However, since peristaltic movements exert perpetual forces on the epithelial cells and underlying tissues, thixotropy should not be neglected. Considering the thixotropy, it was found that Vitrogel achieves a regeneration of its inner structure of 78.9 ± 11.2% in the case of complete destruction, proving that also in the case of higher shear forces Vitrogel would rebuild its network structure to a high extent. In contrast, Matrigel and Peptigel only reached a regeneration of 30.2 ± 11.7% and 42.8 ± 0.3%, respectively. Data found in the literature indicate that the human in vivo ECM shows viscoelastic properties. This implies that after applying stress on the tissue, the deformation decreases in a time-dependent manner, which leads to high regeneration of the tissue, but still slightly affects the structure.^45^ Since Vitrogel has a permanent deformation of only about 20% (vs approximately 60–70%) compared to Matrigel and Peptigel, it better reflects physiological conditions. Besides these similarities to healthy in vivo ECM, Vitrogel exhibits the major advantage of its adaptability with regard to the rheological behavior. It is reported that in diseased states of the small intestine, e.g., inflammatory bowel diseases, the stiffness and elasticity of the tissue changes.^46^ By adding, e.g., collagens, proteins, or other substances present in the ECM to the gel, the properties can be adapted to diseased conditions, which allows on the one hand a change in viscosities and on the other hand an adaptation to molecular occurrences.^47^ The latter is important if biochemical and molecular interactions of the ECM and epithelial/endothelial cells are studied. It is proven that the ECM has an impact on cellular behavior through cell signaling, biochemical sensing, or the interaction with cell receptors, leading to differences in cell proliferation, differentiation, and migration.^46,48^ With this in mind, Vitrogel has been used as an ECM substitute in various studies to test cell differentiation, proliferation, and migration in, e.g., dendritic, glioblastoma, and stem cells.^47,49−51^ For example, the hydrogel was found to allow physiologically relevant dendritic cell chemotaxis in a gastric microfluidic chip without altering dendritic cell activation or viability, as is the case with xenogeneic gels (e.g., Matrigel).^50^ Another study reported that human apical papilla stem cells cultured in Vitrogel showed increased proliferation and differentiation.^51^ This influence could also be observed in the studies presented here. The evaluation of the cell proliferation revealed a significant increase on Vitrogel (i.e., (1.70 ± 0.11)-fold; ***p ≤ 0.001)) compared to a cell control, while Matrigel and Peptigel led to similar results as the cell control. This is probably attributable, first, to the addition of cell culture medium to the gel. Interestingly, proliferation was hardly improved by Matrigel, despite the presence of biologically active ingredients. Second, the polysaccharides as part of the hydrogel enhance cell proliferation, as reported by other research groups.^24,52^ Chen et al. reported an increase in proliferation after the addition of polysaccharides, probably due to the expression of ECM proteins (e.g., collagens) and activation of several signaling pathways.^52^ Another group also presented increased cell proliferation of Caco-2 and HT29-MTX cells through cultivation on synthesized hydrogels.^24^ Although the exact mechanisms are still unknown, the biochemical processes are mainly influenced by the components of the ECM and the interaction of integrins and matrix proteins.^8,48^ For example, the ECM harbors growth factors that have been shown to positively influence cell proliferation.^46^ Moreover, the binding of integrins to glycoproteins, such as fibronectin, laminin, elastin, or collagen, affects cell adhesion as well as proliferation and additionally regulates growth factor receptors. Furthermore, the transcription of genes is triggered, which in turn influences growth, proliferation, and differentiation.^8,48,53^ Based on these results and the results of the rheological characterization, Vitrogel was chosen for further studies as an alternative ECM substitute in the developed small intestinal in vitro model.

As the endothelial cells are part of the deeper tissues of the small intestine and limit the ECM on the opposite site, an endothelial cell layer was incorporated into the model via inverse cultivation. The visualization via confocal microscopy showed a confluent cell layer, confirming the successful cultivation on the basolateral side of the filter over 21 days. Since TEER measurements coincide with the literature,^33^ it was also shown that the endothelial cells developed proper cell–cell connections, which was further proven by staining of occludin as part of the tight junctions (Figure 5J,K,L). This endothelial cell barrier is an important component, since it controls the passage of antigens, drugs, and the commensal gut microbiome.^9^ In addition, endothelial cells play an important role in inflammatory processes as presented by Kasper et al.^54^ Endothelial cells in coculture with Caco-2 cells have been shown to regulate the release of cytokines in vitro and also to contribute to the maintenance of the transepithelial barrier. Moreover, inflammatory mediators have been found not to affect the endothelial side in cocultures, in contrast to monocultures in which cytokines were present on both sides,^54^ proving the importance of including the endothelial cell barrier into small intestinal in vitro models.

Following the endothelial cell seeding, the insert was apically coated with Vitrogel followed by the cultivation of the Caco-2/HT29-MTX coculture on the gel to set up the complete model. Further characterization of the model reinforces the advantages of the hydrogel and the importance of including the ECM based on the effects on the cell system compared to cultivation in blank Transwell inserts. Proceeding to investigate the proliferation rate, the previously obtained results were reaffirmed. The model yielded a statistically significant increase in cell proliferation in the first 14 days of cultivation (day 7: ***p ≤ 0.001, day 14: **p ≤ 0.01). Only day 21 shows a decrease in proliferation and a convergence of the data to the uncoated wells, with no significant difference observed. In line with cell proliferation, the TEER values also rise. Starting with a relatively low TEER value for the model as well as the two control cultures after 7 days, the TEER value increased over the course of 21 days, indicating the formation of a dense cell barrier with high integrity. However, it should be noted that the model exhibits significantly lower values (***p ≤ 0.001) compared to the Caco-2 monoculture. This is due to the fact that goblet cells do not form junctions as tight as a Caco-2 monoculture.^55^ These results are also consistent with those found in the literature. For example, Schimpel et al. investigated the influence of HT29-MTX on TEER levels in coculture with Caco-2 and obtained values similar to those presented here (i.e., 340 ± 6 Ω cm^2^).^16^ Macedo et al. also reported similar findings.^27^ Nevertheless, the model yielded even lower TEER values than the coculture without gel. This was also observed by Li et al. while culturing intestinal cells on a collagen-hydrogel.^56^ One reason for this could be that the gel affects the conductivity between the apical and basolateral sides and, thus, also changes the resulting resistance. Despite the decreased values, the physiological TEER values of the small intestine range from about 24 to 66 Ω cm^2^.^57^ Taking into account different measurement methods and the fact that Caco-2 cells are known to express high levels of tight junctions and forming a tight barrier, we are approximating the physiological occurrences with the model.^58^

In parallel to the increased cell proliferation, accelerated differentiation of Caco-2 cells was also detected in the model. Therefore, ALP release was determined to confirm cell differentiation with and without Vitrogel, using the Caco-2 monoculture as cell control. As expected, the monoculture yielded the highest ALP secretion. Due to the lower number of Caco-2 cells in the coculture, only a lower amount of ALP was detected regardless of the cultivation with or without gel. Similarly, Schimpel et al. also observed decreased ALP activity in the cocultivation of Caco-2 and HT29 MTX cells.^16^ The cultivation in uncoated wells of the coculture revealed its highest ALP release on day 21. In contrast, the model has already reached 95.0 ± 15.9% (n.s.) of ALP production on day 14, decreasing to 83.0 ± 1.3% (*p ≤ 0.05) on day 21. Since ALP is a brush border enzyme, the results indicate that the enterocytes form a functional brush border by day 14 in the model.^34^ These results also correlate with those of cell proliferation, as the data on days 7 and 14 showed a significantly higher proliferation in the model compared to the coculture. Another indicator for the faster differentiation of the cells is the formation of tight junctions and microvilli structures. Although the formation of tight junctions was observed in all cultures after 14 days independent of the ECM layer, the images of the model indicate that cells grow in a more organized structure and that the Caco-2 cells in the model present microvilli already by day 14. Natoli et al. reported a comparable formation of microvilli only after 21 days.^59^ The combination of these data with the ALP results indicates fully developed Caco-2 cells in the model. Another important characteristic of the small intestine is the mucus layer. The mucus layer produced by goblet cells not only hinders diffusion of unwanted substances and bacteria but also hosts the gut microbiome. Since it completely covers the enterocytes, it additionally plays a pivotal role in drug delivery.^60,61^ The results obtained are comparable to those found in the literature, e.g., Pham et al. reported the formation of confluent mucus layer covering the enterocytes using the same coculture as presented here (7:3 coculture of Caco-2 and HT29-MTX cells).^62^ The staining shows an evenly distributed mucus layer in the HT29-MTX monoculture as well as in the model from day 14 onward. Since the coculture in blank wells only exhibit gel fragments after 21 days, it can be assumed that Vitrogel enhances the mucus production of the HT29-MTX cells. In that regard, the confluent mucus layer also explains the reduced ALP levels in week 2, as the ALP release is likely impeded by the mucus-covered enterocytes.

As a last parameter, the permeability of different permeable substances was investigated. As expected, no *P*_app_ value could be determined for FITC-dextran, since this substance is considered a nonpermeable drug due to its high molecular weight. On the contrary, the *P*_app;A→B_ values detected for antipyrine indicate promising results. The model consistently shows a high permeability, with a peak already on day 14 (i.e., (33.64 ± 5.13) × 10^–6^ cm/s). This result is consistent with data obtained in ex vivo experiments. Rozehnal et al. examined human small intestinal tissue mounted in an Ussing chamber to test permeability. They reported values in the physiological range (i.e., (32.7 ± 4.69) × 10^–6^ cm/s), which coincide with our results.^37^ The *P*_app;A→B_ of atenolol are also consistent with data obtained in ex vivo studies using excised small intestinal tissue.^38^ Furthermore, this suggests that neither the ECM nor the endothelial cell layer hinders the paracellular transport of the drug. Interestingly, it was also found that the B → A permeability was higher, indicating the activity of P-gp transporters and an efflux-mediated transport of the drug.^39^ The P-gp activity could also be confirmed by the determination of rho123 permeability. *P*_app;A→B_ could not be calculated, as the obtained rho123 concentrations were below the LOD. The B → A permeability also shows low values compared to Volpe et al.^30^ However, the higher values obtained by Volpe could be attributed to the higher expression of P-gp transporters in Caco-2 monocultures. Since the model consists of a coculture of two cell lines and exhibits a lower TEER, a decrease in the *P*_app;B→A_ is to be expected.

## 5 Conclusion

State-of-the-art in vitro models mimicking the small intestine usually consist of a cell monolayer without considering the ECM and endothelial cells. In this study, a cell model incorporating the ECM and the basolateral endothelial layer was developed and carefully characterized. The ECM substrate Vitrogel, a ready-to-use polysaccharide hydrogel produced synthetically in a controlled environment, has emerged as a suitable alternative to animal-derived gels. The gel not only increased cell proliferation but also enhanced the differentiation of the cells, while enabling an unhindered permeation of drugs. In combination with the epithelial as well as the endothelial cells, representing the basolateral blood vessel compartment, a full differentiation within 2 weeks with regard to the ALP release as well as the tight junction, mucus, and microvilli formation was achieved. Permeability studies confirmed similar values to ex vivo occurrences, proving that the gel not only improves cell proliferation and differentiation but also does not impact the permeability of the drugs. One major advantage in the use of Vitrogel is also its adaptability to desired biochemical and rheological properties by incorporating collagens, laminins, or biologically active molecules such as growth factors. In combination with epithelial and endothelial cells, this model can further be used for the investigation of, e.g., inflammatory processes that show altered viscoelastic behavior and biologically active molecules. In addition, the model raises many possibilities in terms of drug or drug delivery system testing and enables a step toward animal-free cultivation, resulting in fewer divergent results compared to, e.g., Matrigel or rat collagen I.
